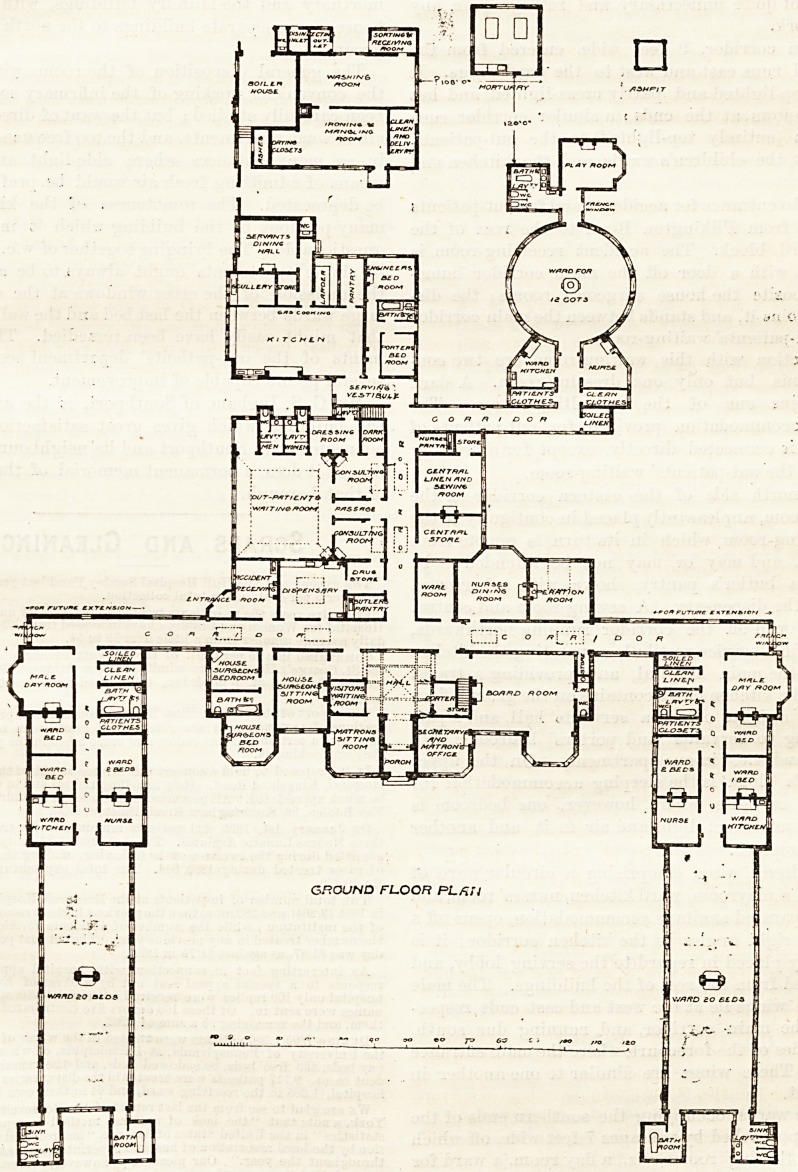# Hospital Construction

**Published:** 1896-11-07

**Authors:** 


### The Institutional Workshop.

### HOSPITAL CONSTRUCTION.

### NEW INFIRMARY, SOUTHPORT.

This building, opened in September of last year, as seen from the south or principal front, occupies three sides of a court-yard, measuring about 170 feet across; the administration block of two floors faces the open end, and the wards and their adjuncts are single-storey buildings, and form the two sides. The administrative block is entered in the centre by a vestibule, leading to a top-lighted central hall; on the right are grouped the secretary's and matron's offices, the board-room, and a porter's room, and on the left a matron's sitting-room, a visitors' room (served by a borrowed light), and a house surgeon's sitting-room. A staircase leads from the hall to the matron's bedroom and bedrooms for sixteen nurses on the first floor, where a nurses' sitting-room is also provided, with a box-room, three bathrooms, and three w.c.'s. The latter open directly off the central corridor, but one of the bathrooms has no direct light or air to it. Returning to the ground floor a door from the house surgeon's sitting-room opens into a passage (apparently with no light at all to it), giving access to the house surgeon's bedrooms, bathroom, and w.c., the two latter in one apartment, and it is to be noticed that the baths here and generally, except those for the patients' use, are shown with casings, an arrangement quite unnecessary and retrograde in any hospital work.

The main corridor, 9 feet wide, entered from the central hall, runs east and west to the ward wings. It is partly top-lighted and partly cross-lighted, and has French windows at the ends; a similar corridor runs northwards (entirely top-lighted) to the out-patients' department, the children's wards, and the kitchen and offices.

A separate entrance for accidents and for out-patients is arranged from Pilkington Road to the rear of the western ward block. The accident receiving-room is well placed, with a door off the main corridor immediately opposite the house surgeon's rooms; the dispensary adjoins it, and stands between the main corridor and the out-patients' waiting-room.

In connection with this waiting-room are two consulting-rooms, but only one dressing-room. A dark room adjoins one of the consulting-rooms. The sanitary accommodation provided for out-patients of both sexes is connected directly, except for a lavatory lobby, with the out-patients' waiting-room.

On the north side of the eastern corridor is the operating-room, unpleasantly placed in contiguity to the nurses' dining-room, which in its turn is remote from the kitchen, and may or may not be intended to be served by a butler's pantry, shown with a borrowed light from the dispensary. A sewing-room and central store-room open off the corridor running northwards, which ends in a service vestibule, separating the kitchen block from the main hospital, and providing a trades entrance. The kitchen block contains on the ground floor the kitchen and its offices, a servants' hall, and a passage leading to servants' and porters' bedrooms with bathroom and w.c. (in one apartment). On the upper floor of this block is the sleeping accommodation for the women servants, where, however, one bedroom is shown without direct light and air to it, and another without a fireplace.

The children's wing, comprising a circular ward of twelve cots, a playroom, ward kitchen, nurses' room, and properly separated sanitary accommodation, opens off a corridor at right angles to the kitchen corridor; it is conveniently placed in regard to the serving lobby, and well removed from the rest of the buildings. The male and female wings lie at the west and east ends respectively of the main corridor, and, running due south, form the sides of the forecourt, where the main entrance is placed. These wings are similar to one another in arrangement.

The large wards occupying the southern ends of the wings are approached by passages 7 feet wide, off which open the following rooms, viz., a day room, a ward for two beds, and two wards for one bed each, a bathroom and w.c. (in one apartment), stores for soiled and clean linen, and a ward kitchen and nurses' room adjoining the large ward.

The large wards are arranged for twenty beds each, measuring 83 feet by 26 feet, with sanitary accommodation and bathrooms in separate blocks at their southern ends connected with the wards by cross-ventilated lobbies. They are warmed by central stoves placed at right angles to the axes of the wards, are lined with tinted-glazed bricks, and the floors laid with oak parquetry. The corridor floors are finished in mosaic. The mortuary and the laundry buildings, with the boiler-house, form separate buildings to the north of the main group.

The general disposition of the rooms with regard to the convenient working of the infirmary seems to have been carefully studied; but the want of direct light and air to some apartments, and the too free use of top-light in so many instances where side-light and an easy means of admitting fresh air would be preferable, is to be deprecated. The remoteness of the kitchen from many portions of the building which it must serve is questionable. The bringing together of w.c.'s and baths in single apartments ought always to be avoided, and the omission of the cross-windows at the ends of the large wards between the last bed and the wall is a defect that might easily have been remedied. The arrangements of the out-patients' department seems to leave several points capable of improvement.

Mr. C. S. Ingham, of Southport, is the architect for the building, which gives great satisfaction to those connected with Southport and its neighbourhood, forming, as it does, a permanent memorial of the centenary of that borough.

## Figures and Tables

**Figure f1:**